# Positive facilitators of diabetes management in emerging adults with type 1 diabetes—A qualitative analysis of blogs

**DOI:** 10.1002/edm2.161

**Published:** 2020-06-19

**Authors:** Clea Bruun Johansen, Frans Pouwer, Henning Beck‐Nielsen, Mette Juel Rothmann

**Affiliations:** ^1^ Steno Diabetes Center Odense Odense University Hospital Odense Denmark; ^2^ Department of Psychology University of Southern Denmark Odense Denmark; ^3^ Department of Endocrinology Odense University Hospital Odense Denmark; ^4^ Centre for Innovative Medical Technology Odense University Hospital University of Southern Denmark Odense Denmark

**Keywords:** diabetes mellitus, qualitative analysis, Type 1, young adult

## Abstract

**Introduction:**

Emerging adults (18‐30 years) with type 1 diabetes must manage a demanding chronic illness as well as navigate a life phase full of instability and transitions. Clinical care for this age group remains a challenge. An improved understanding of psychological facilitators of diabetes management may contribute to optimized clinical care to this age group.

**Aim:**

To explore which individual strategies facilitated emerging adults’ diabetes management and what kind of support they regard helpful from peers, family and healthcare providers.

**Methods:**

Qualitative analysis of web blogs. We identified personal blogs by emerging adults with type 1 diabetes through a search at the websites for diabetes associations in Denmark, UK and the USA, a snowball search at identified blogs and an internet search (Google). Blog posts from approximately the last year were analysed with thematic analysis as described by Braun and Clarke.

**Results:**

We included 16 blogs from UK, the USA, Australia and Denmark, focusing on blog entries from 2017 to 2018. Several psychological facilitators of management of type 1 diabetes were identified. Positive individual strategies involved: developing a balanced approach to blood glucoses, sharing diabetes with peers and making space for emotional reactions. Supportive involvement from peers, family and health care providers included: normalization, emotional backup and a nonjudgmental attitude.

**Conclusion:**

Diabetes management in emerging adulthood can be facilitated by several individual strategies as well as by supportive involvement from peers, family and health care providers. It is worthwhile to further investigate how individual strategies as well as supportive involvement can be promoted in diabetes care.

## Abstract 2

A qualitative analysis of personal blogs written by emerging adults with type 1 diabetes documented psychological strategies that facilitated diabetes self‐management.

## 1 INTRODUCTION

‘Diabetesgeek’, ‘My bitter sweet life’, ‘Flawless diabetes’ and ‘The pumptastic scot’ are some of the catchy titles of internet blogs—short for weblogs—written by young adults with diabetes. The blog themes are diverse, but often the author posts personal stories from life with diabetes, such as concrete everyday struggles with the daily diabetes management.

These blogs can therefore potentially inform about ‘the impact of having diabetes’ and ‘diabetes management issues’ in a notoriously challenging age group, often characterized by suboptimal diabetes management, ambulatory nonattendance as well as increased risk for health complications and premature death.^1^, ^2^

It is fruitful to consider young adults’ diabetes challenges in the context of psychological development.^3^ Contemporary psychological theory describes the ages from around 18 years and up through the twenties as a particular developmental phase, ‘emerging adulthood’.^4^ The phase is usually more tumultuous than later phases of adulthood and some of the key features are identity exploration, self‐focus and an ambivalent dependency on parents paired with a search for autonomy.^4^

Still, the dynamics of diabetes self‐management during emerging adulthood is far from fully understood.^1^, ^2^, ^3^ A better understanding of positive facilitators of diabetes management may contribute to fill the gap between our recognition of the management challenges in emerging adulthood and how to practically intervene to support better management.

Current psychological and social research on diabetes in emerging adulthood primarily consist of relatively few qualitative interviews and questionnaire studies.^5^, ^6^ Such studies provide important information, but also suffer methodological limitations, such as low response rate in questionnaire studies and response bias (ie the tendency for respondents to provide socially acceptable responses). Furthermore, both questionnaire studies and interview studies place the emerging adult in a somewhat passive role as respondents to research questions. Personal diabetes blogs offer alternative access to emerging adults’ voices. The blog content actively reflects what goes on in the young people's own lives and may provide spontaneous and self‐selected information.

In short, personal blogs bear resemblance to traditional diaries, displaying entries, or posts, in reverse chronological order. The style is often informal and accompanied by pictures from everyday life. Personal blogs are often publicly accessible at the Internet and offer the opportunity for contact with audience through comments.^7^, ^8^ Several motivations for blogging have been identified; for example documenting one's life, expressing deeply felt emotions, providing opinions and forming and maintaining community forums.^8^, ^9^, ^10^ For adolescents and young adults, playing with self‐presentation and identity exploration has been suggested as central motivators.^11^, ^12^ Bloggers come in all ages. Still, the activity of blogging seems to have a special appeal to younger age groups. A report tracking characteristics of bloggers showed that 54% of bloggers are between the ages 18 and 29.^13^ As such, blogs seem to represent a natural arena for expression for emerging adults.

Analysis of blogs represents a novel approach and few diabetes studies have been based on blogs.^14^, ^15^, ^16^, ^17^, ^18^, ^19^ No previous diabetes blog studies have focused on emerging adults, even though the age group was included in some of the studies.^14^

In this study, our aim was to explore which individual psychological strategies, and what kind of social support, were perceived as helpful by emerging adults in dealing with their management of type 1 diabetes. We hoped that this information would be helpful for the future development of age‐targeted clinical support tools for emerging adults with diabetes.

We focused on two main research questions: (1) Which positive individual strategies were expressed by emerging adults as facilitating diabetes management?; and (2) What kind of support from peers, family and health care providers were regarded as being helpful by emerging adults to facilitate management of diabetes?

## 2 METHODS

We conducted a qualitative analysis of blogs written by emerging adults with type 1 diabetes.

### 2.1 Framework

There is no firmly established methodology for how to identify and select blogs or how to extract and analyse content.^20^, ^21^, ^22^, ^23^ In this study, we applied thematic analysis, which has previously been used for analysing blogs authored by persons with diabetes in relation to topics such as eating disorders and exercise.^14^, ^15^

### 2.2 Criteria for inclusion of blogs

Blogs were included that were authored by an individual emerging adult (including all phases of emerging adulthood: ages 18‐30) with type 1 diabetes, if the blogs contained a broad spectrum of themes on life with diabetes (>3 themes addressed), if they were in Danish or English, if they were publicly accessible and if the blog had been active within the last two years.

Blogs were excluded if it was not possible to determine the age of the author or if the person with diabetes wrote only about one or two themes of diabetes, such as marathon running.

### 2.3 Search strategy and sampling

The search was conducted from June 2018 to September 2018. Three search strategies were used to identify eligible blogs:

- A search at the websites for the main diabetes associations in Denmark, UK and the USA to identify recommended blogs (inspection of the list of content and search for the terms ‘blog’ and ‘blogs’.
- A snowball search in blogrolls (list of links to other blogs) at the identified blogs authored by emerging adult with type 1 diabetes.
- A supplementary Google search that involved the central terms: blogs, emerging adulthood and diabetes using different synonyms (search strings: young adulthood AND blog AND diabetes, emerging adult AND blog AND diabetes)

The aim was to include at least 15 blogs. This number was pragmatically chosen as the anticipated minimal number of blogs required to achieve saturation of the main themes addressed.

### 2.4 Data analysis

All textual blog content was copied into Word files, dates for copying were noted and all content was imported into NVivo [state product version, eg NVivo 12 Pro] qualitative analysis software (QSR International).^24^

We used a deductive approach to focus on features pertaining to the two research questions: individual strategies for diabetes management in emerging adulthood and support from peers, family and healthcare providers. The overarching principle for the analysis was to obtain relevant information from each blog regarding our research questions. The concrete procedure for the analysis was inspired by Braun and Clarke (2006) in the following five phases ^25^:

In phase 1, all blog content was read to get familiar with data and to obtain a general overview of conceptual patterns resulting in a tentative list of ideas and themes. In phase 2, we identified units of meaning, relevant to the research question, and formulated initial codes. For each blog, we focused on the period of the last blog entry and approximately one year back. In blogs with low blogging activity, we extended the period. For two blogs, the period was extended with up to 6 months, and for one blog we extended with 2 years. In blogs with high blogging activity, we reduced the period. For two blogs, we reduced the period with up to 6 months. The exact timing, and resulting number of blog entries analysed, was decided on a case‐by case basis, resulting from our assessment of whether saturation regarding our research questions was reached (Table 1; Box 1). In phase 3, relationship between codes, themes and levels of themes were investigated, resulting in a collection of candidate themes. In phase 4, themes were refined, through investigating meaningful coherence within themes and clear distinction between themes. Phase 5 consisted in defining overarching themes and labels to capture the overall essence of what each theme was about. Accordingly, within each theme, the final sub‐themes (themes within the themes) were also formulated (Table 2). This process of formulating, refining and revising codes and themes proceeded until saturation, which was determined based on no new themes being uncovered.^25^

**TABLE 1:** Blog and blogger characteristics^a^

Blogger demographics	Value
Female gender, n (%)	15 (94)
Country	
UK	7
USA	4
Australia	2
Denmark	2
Canada	1
Age in years^b^, mean (SD), range	23,9 (3,4) 18‐29
Blog features	
Years with active blogging^c^, mean (SD), range	4,6 (2,4) 1‐9
Blog activity (number of entries, last year), median, range	9, 1‐118
Pictures (number of pictures per blog, last year)	
0‐9	7
10‐49	6
>49	3
Median word count per entry per blog (last year)^d^, mean (SD), range	607,4 (245,5), 340‐1279,5

^a^ See box for links to blogs, active when data was collected.

^b^ age is approximate, as it was deduced based on blog content.

^c^ years of blogging is based on year of the first entry until year of data analysis.

^d^ word count per blog entry differed, and we report the mean of the 16 medians.

**TABLE 2:** Analysis process with examples^a^

Phase 1: Tentative themes were formulated after an in‐depth reading of blog content	Phase 2: Units of meaning^b^ were identified and initial codes formulated		Phase 3: Candidate themes were investigated	Phase 4 and 5: Themes and subthemes were refined and overarching themes and sub‐themes defined
Tentative themes Imperfect diabetes management Struggling is normal Sharing stories Sharing frustrations Blood sugars is all‐pervasive Part of something bigger Transcending individual diabetes Solidarity	Units of meaning *‘Blood sugars will rise and fall, I haven't come across anyone whose blood sugars remain the same. Stress will come and go in many different forms and life will keep on passing by. Maintaining the charge in your battery will always be hard, but never impossible’*. (Kayla's life notes) *‘This conference, this room full of women who shared this lived experience deeply and daily—this is where I came to feel known’* (Coffee and insulin)	Initial codes Accept that blood sugars rise and fall and recharge batteries Benefits from reaching out for others	Candidate themes Find a balanced way to cope with fluctuating blood sugars Reach out for others	Overarching theme and sub‐themes Positive individual strategies that facilitate diabetes management A balanced approach to blood glucoses Share diabetes with peers

^a^ Examples of flow from tentative themes to overarching themes.

^b^ Units of meaning are phrases or sentences that carry a set of perceptions or an idea

#### BOX Links to included blogs^a^

**Table:** 

Amybetic	https://amybetic.wordpress.com/
Coffee and Insulin	http://www.coffeeandinsulin.com/
Diabetesgeek	http://diabetesgeek.blogspot.com/
Flawless diabetes	http://flawlessdiabetes.com
	(https://flawlessdiabetes.tumblr.com/)
Iced coffee and insulin	https://icedcoffeeandinsulin.wordpress.com/
Insulin Pens don't have ink	http://insulinpensink.blogspot.com/
Kayla´s life notes	http://www.kaylaslifenotes.com/
Min diabetes [My diabetes]	https://www.mindiabetes.dk/
My bitter sweet life	http://elshuckle.blogspot.com/
Sweet and sour – the type 1 diabetes rollercoaster	https://adiabeticsrollercoaster.blogspot.com/
Teapot diabetic	http://teapot8909-diabetic.blogspot.com/
The pumptastic scot	http://www.pumptasticscot.co.uk/
Type1writes	https://www.type1writes.com/
TypeOnederful	https://typeonederful.com/
Vicki's notebook	http://vickisnotebook.blogspot.com/
What the ´betes	http://whatthebetes.com (domain expired)

## 3 RESULTS

We included 16 blogs (Table 1).

### 3.1 Descriptive overview

Authors were female in 15 blogs (Table 1). 15 blogs were in English (seven were from UK, five from the United States, two from Australia and one from Canada). Two blogs were in Danish. Bloggers’ age ranged from 18 to around 29 years. The blogs all provided a vivid picture of everyday life with type 1 diabetes for emerging adults, with accounts of small and big, joys and victories, setbacks and frustrations. The style and content varied to some extent, some blogs mainly reported descriptively on everyday life whereas other blogs were more emotionally charged with personal reflections on life, meaning etc Some blogs used a humoristic tone, also when describing challenges and emotional difficulties. All blogs contained personal pictures, but some to a greater extent than others.

The analysis of blog content was organized under two main themes: (1) Positive individual strategies that facilitated diabetes management; and (2) Support from peers, family and healthcare providers. Each main theme included several sub‐themes described below.

#### 3.1.1 Positive individual strategies that facilitated diabetes management

Across all blogs, it was a dominant theme how to deal with the all‐invasive and continuous demand for blood glucose monitoring and the never‐ceasing risk for ‘highs’ and ‘lows’ (hyperglycemia and hypoglycemia). All blogs contained meticulous descriptions of diabetes management in all sorts of situations; for instance, how to monitor blood glucose during fitness, how to eat at Christmas, how to navigate diabetes setbacks in new college settings, how to tidy up all the diabetes devices, strips and cannulas, and so forth. From these descriptions, it emerged how blood glucose amalgamated with most other aspects of the young adults’ daily living and that it consequently was crucial to develop a balanced approach to blood glucoses. A ‘balanced approach’ allowed for continuous monitoring *as well* as for having bad days, for making mistakes, for not being too tough on oneself and for not getting sucked into self‐blame due to blood glucose numbers.

The need for developing a balanced approach was important, also because blood glucoses could inflict negatively on self‐worth, as stated in numerous blogs. Accordingly, several bloggers reflected on the importance of finding a personal approach to immediate blood glucose numbers that enabled to feel fundamentally okay despite single numbers. One blogger formulated it like this:

It’s so easy to get caught up in these numbers, and so easy to forget that we’re all just human; perfectly imperfect; living, breathing, fighting and trying.

We’re so much more than just a number, and sometimes I think we need to remind ourselves of that. (Amybetic)

Developing a balanced approach to blood glucose also entailed to deal with feelings of guilt or self‐blame due to blood glucoses rising and falling. One blogger described that there is a lot of self‐inflicted guilt related to diabetes management and emphasized the importance of telling yourself that you are doing a good job:

I also remind myself that along with those amazing tools, comes some work on my end of things, something that technology isn't capable of helping, which is my mind and ability to say to myself, “you're doing a good job’. (Kaylas life notes)

Likewise, many bloggers retold all the other things they have achieved in life, regardless of blood glucoses. Accordingly, some blogs contained lists of life achievements and personal interests, other than diabetes.

### 3.2 Share diabetes with peers

Another consistent strategy in all blogs to facilitate diabetes management was to share diabetes. This strategy was embodied in the blogging activity as such, enabling the young adults to reach out to others, both to a random internet audience, to other family members and particularly to young adult peers with diabetes. It was especially rewarding to share with other young people with diabetes. One of the main reasons was that it alleviated loneliness with diabetes:

If you are going through a frustrating T1D time, or feeling like you just can’t do it anymore, I encourage you to reach out to the T1D community online, because you are not alone. (Iced coffee and insulin)

Most bloggers also described how they reached out for peers in real‐life encounters. Participating in diabetes meetings and events and doing voluntary diabetes work was thus described as rewarding in several blogs.

The diabetes issues that the young adults shared, online as well as in real life, were on many levels. Some bloggers primarily shared their concrete frustrations with blood glucose monitoring and everyday annoyances and practicalities about devices, strips and foods. This provided them with an opportunity to learn from each other about practical diabetes management and it also gave them the opportunity to take on the role as experts on diabetes management. As illustrated by this quote, it just felt nice to not be the only one who must manage diabetes:

…it really is nice to whack out your finger pricker alongside someone else doing the same thing for once. (What the ´betes)

Also, they were often used as an outlet for anxieties, deep frustrations and reflections on the emotional tolls from living with diabetes as a young adult. Thus, several bloggers formulated that it was a relief to not be alone with all these difficult emotional reactions. Through sharing diabetes with a peer group, the bloggers described that they gained something positive; they experienced a valuable emotional bond to some great people. One blogger consequently recommended:

Find your tribe, love them hard. (Vicki’s notebook)

### 3.3 Tap into a higher meaning

In all blogs, the connectedness to other young people seemed to carry intense meaning. This taps into a related strategy, also found in most blogs, namely, to apply meaning to diabetes management that transcended the immediate blood glucose numbers and made the day to day hassles worth it.

Attributing meaning was done in various ways, such as describing life with diabetes as a journey, quoting famous wisdom words, pondering upon the personal strength that sprang from diabetes, belonging to the diabetes peer group and being loved and supported by family through tough times with diabetes.

Several bloggers contemplated on exciting life opportunities that rose from diabetes, such as talking at the parliament and becoming a diabetes advocate. Others valued the personal strength they have developed from managing diabetes. One blogger formulated it like this:

…as unfortunate as living with Type 1 Diabetes is it has made me a better person and I truly believe this. (My bitter sweet life)

Using humour was another way to rise above the immediate day to day troubles. In this sequence, the blogger jokily described her personal journey from suboptimal diabetes management to a meaningful endeavour allowing her to stay healthy:

Fast forward a couple of years and a little bright lightbulb has clicked in my head. Yep – diabetes is rubbish, and whacking out my thigh and a needle in front of people I met 10 minutes ago isn’t always ideal – but I love my body, and actually – I want to be healthy, and for a long, long time! SO. I’ve snapped out of my mediocre diabetes management and upped my game. (What the ´betes)

### 3.4 Make space for the emotions

The importance of the emotional side of diabetes was articulated in many blogs, and it was frequently described how managing diabetes depended on the emotional state. As one blogger stated, the emotional acts of diabetes weighed much heavier than the practical acts (Vicki's notebook). The emotional state was thus a prerequisite for managing diabetes properly:

I can test my blood sugar, but whether or not I act on the numbers my meter shows me very much depends on where my head's at. (Vicki’s notebook)

For many bloggers, it was important to be able to talk openly about emotional issues:

Living with diabetes makes us even more prone to facing mental or emotional issues, and talking about these issues often makes them easier to deal with’. (Type1writes)

Most blogs continuously addressed everyday emotional reactions, such as frustrations, loneliness and bad conscience. Several blogs also described how serious emotional problems, such as anxiety and depression, could sabotage diabetes management. In these cases, it was important to ask for help:

Anxiety is not less than depression, it's not something to be pushed aside as a quirk. Don't let anxiety be your silent saboteur, ask for help. (Sweet and sour)

#### 3.4.1 Support from peers, family and healthcare providers that facilitated diabetes management

Support from both peers, family and healthcare providers was mentioned in all blogs. In some blogs, support from peers, family and healthcare providers was characterized through recurrent descriptions of situations where they received support. The tone and language used for describing support were often emotionally charged, underlining the significance of receiving help from other people. Also, many blogs contained meta‐reflections on the value of receiving support and some bloggers directly addressed those who supported them to let them know that they were treasured:

So this post is dedicated to you ‐ my friends, my family, my extended (healthcare) family ‐ it's you I value the most! (Coffee and insulin)

### 3.5 Normalization and understanding from peers

The most frequently and detailed descriptions were of support from peers. Several bloggers contemplated on the reasons why support from other young people was so particularly helpful. It was for instance argued that peers provided a special kind of understanding based on their own intrinsic knowledge about diabetes. Often, support from peers revolved around going through the same highs and lows and having to live with the continuous demands for monitoring the blood glucose:

Everyday at this camp, I was surrounded by people who also checked their blood sugar, who also had hypos, who also struggled Day to day with type 1 diabetes, and it almost normalised these things. For once, I felt normal, not ill or different. When we went swimming, I wasn't the only one with a cannula in or with scars from countless needless. It felt amazing. (The pumptastic scot)

As reflected in the sequence above, normalization was a key feature in support from peers. This pertained to normalization of having a chronic illness and, maybe even more important, it pertained to normalizing the emotional struggling related to managing this chronic illness. It was recurrently reported that it was a relief that other young people found it hard to control their blood glucose. Peers also provided normalization by being at the same life phase:

Nothing beats understanding, particularly from those that are at a similar stage of life as me (twenties, wading through life not really having a clue where you're at, who you are or what you're doing, and T1D is along for the ride… (Vicki’s notebook)

### 3.6 Persistence, encouragement and love from family

Support from family was highlighted by several bloggers. Many bloggers emphasized the significance of their family members’ ability to carry on when the young adult lost courage. Especially, the continuous help from mothers was praised and several blogs contained letters dedicated to mothers. These letters were highly emotive and underlined the significance of support from someone who loved you, who was persistently by your side in emotionally tough situations and who provided ongoing encouragement:

You held me through it all. From that first alarming appointment, you supported me with unwavering steadiness. You gave me a reassuring feeling that no matter what was going to happen, that your support was everlasting and that you would be by my side throughout this journey. Your calmness was admirable. Your ability to make me feel like I was not going through this all alone was so important. You made me laugh in those moments when I was uncontrollably crying. You sat with me even when I did not want to talk. (Iced coffee and insulin)

Several blog posts also described how family helped with practicalities, fetched supplies, drove to the hospital or picked up test strips. This kind of help not only made diabetes easier on a practical level but also made the young people feel cared for. Continuous practical help from family thus made diabetes more manageable on an existential level.

### 3.7 Nonjudgmental and individualized support from healthcare providers

Support from healthcare providers was described in several blogs. Some bloggers reported on specific situations, where they had received important help, for instance in relation to blood glucose management. One blogger explained how she had received valuable help to monitor less tight, as she was obsessing about her numbers to a degree that compromised life quality (Insulin pens don't have ink).

In general, it was important for the young adults that healthcare providers truly comprehended the multifaceted nature of blood glucose monitoring and that they were able to individualize their approach. Some bloggers pointed out that it could be very stressful to go to checks at the hospital and that this stress could be alleviated when healthcare providers did not judge them by blood glucose numbers. In the following sequence, one of the bloggers contemplated on her next appointment at hospital:

My next HbA_1c_ check is in July, when I've got my annual review (so eyes, feet, blood, the works.) Yes, I will say here and now that I am nervous about it (and, yes, I know I have months before that appointment) but I'm so used to being defined by my number. Even after almost 2 years under Norwich care, I'm still not used to their attitudes towards it all. However, as nervous as I am about it, I know that I will not be "judged" for it, and I will be given help and advice, and I will work towards a "good" hba1c. (Vicki’s notebook)

Several bloggers valued support for emotional issues. For instance, help to cope with anxieties and help to handle obsessive behaviours related to blood glucose monitoring. However, some bloggers highlighted that emotional problems were often overlooked and that healthcare provider resources were scarce in this area (eg Type1writes).

Accordingly, several bloggers emphasized that they wished to be met as individuals and that they wished support for both the mental and the physical side of diabetes. Likewise, they wished that the healthcare providers understood that being in the life phase of emerging adulthood posed specific demands. One blogger thus directly formulated how she would like to be addressed by healthcare providers:

If I could re create the conversation between health care provider and myself, I'd make it something like this: “We understand that you are living a busy life with school, your social life and diabetes. Please let us know if there is any way we can support you in your diabetes management so that we can help you get to a better place both physically and mentally with your diabetes.” (Kayla’s life notes)

## 4 DISCUSSION

Sixteen blogs authored by emerging adults living with diabetes were analysed to identify key psychological facilitators of management of type 1 diabetes, including individual strategies as well as support from peers, family and friends. Positive individual strategies involved to develop a balanced approach to blood glucoses, to share diabetes with peers, to attribute a higher meaning to diabetes management and to make space for the emotional reactions. Supportive involvement from family, friends and healthcare professionals was highly valued in all blogs and included peer understanding and normalization, persistent emotional backup and encouragement from family and from healthcare providers; trust, a nonjudgmental attitude and a true comprehension of the multifacetedness of blood glucoses in the emerging adulthood.

These findings are relevant to consider in relation to previous studies. Anderson and Wolpert discussed factors that may determine young adults’ success in independent diabetes management and argued that developmentally based principles for clinical practice are important, such as building a relationship with the young adult and working with the family.^26^ Also, a systematic review of barriers and facilitators for clinic attendance after young people's transition to adult care concluded that care delivery should be sensitive to the developmental characteristics of young adults.^27^

Interestingly, our focus on blogs also taps into an increased recognition of the significance of online activities among people with diabetes in all ages. Hilliard et al (2015) thus described the rapidly growing Diabetes Online Community (‘DOC’) and recommended further research into positive impacts.^23^, ^28^ In a recent review, Oser et al emphasized that there appears to be significant ability to turn to the DOC to explore psychosocial needs and outcomes in people with diabetes.^17^

Even if we know of no previous blog‐based diabetes studies primarily about emerging adults, blogs were assessed useful in studies of other populations. This was for instance the case in Staite et al’s study about diabetes and eating disorders and in Oser et als investigation of exercise in adults with type 1 diabetes.^14^, ^15^

The blogs included in our study can be read through the lens of the developmental needs in emerging adulthood.^4^ One essential task in this age phase is to explore one's identity and find out ‘who am I as an adult?’ and the blogs demonstrated that diabetes was not managed in parallel with that. Rather, it seemed, diabetes management was fundamentally entangled with this identity task.

An encompassing strategy identified in all blogs was to develop a balanced approach to blood glucose and to the inevitable fluctuations. This was crucial, as managing blood glucoses were an all‐invasive endeavour in the young people's life and demanded enormous energy. The demands did not solely have to do with the ‘practical acts’ of diabetes, as named by one blogger, but more with the way blood glucose merged with most other aspects of life; with going to college, with dating, with moving away from home, when eating out, etc. A key aspect of the balanced approach was to remind oneself that ‘you are doing a good job’ despite ‘highs’ and ‘lows’ and to avoid being sucked into self‐blame and guilt.

Accordingly, support from peers, family and healthcare providers that facilitated an individualized approach was helpful. Key features of support were normalization of struggling with blood glucose struggles, ongoing encouragement and a nonjudgmental attitude. This corresponds well with a recent review on parental support for emerging adults with type 1 diabetes, reporting that a noncontrolling approach from parents is beneficial for young people's diabetes management.^29^

Sharing diabetes with others was a strategy that could be identified in all blogs. Especially, it was rewarding to share diabetes with emerging adult peers. They shared practical as well as emotional aspects of diabetes. Thus, sharing diabetes gave the young people a crucial feeling of not being alone and of being accepted, despite struggles with blood glucose monitoring and emotional reactions. By sharing, it can be argued, the emerging adults transferred diabetes management from the individual sphere, often full of self‐blame, to a collective sphere, where frustrations and emotional reactions were legitimate and even the norm.

It is interesting to consider the strategy of sharing in the light of psychological theory about belonging, underlining that it is a basic human need to belong to a group and that it is a central driver of motivation to be valued and accepted by others.^30^ Interestingly, belonging is already formalized in many self‐help groups, including well‐known group programs such as Alcoholics Anonymous (AA) and Weight Watchers (WW).^31^

Notably, the themes identified in our study share some similarities with those found in some other blog studies, where giving and receiving peer support was a constant regardless of age. Caregivers to children with diabetes became resilient through peer support, adolescents used social media to seek peer interactions, support and advice etc.^17^

The benefits from peer support are also highlighted outside the online sphere, hence, a recent survey of emerging adults and adolescents demonstrated that peer support predicts treatment adherence in these age groups.^5^ Our study added to this, suggesting that peer support facilitated diabetes management both because it enabled the young people to share practical experiences and be diabetes experts, and even more important, it provided them with a meaningful space for their diabetes management struggles.

Attributing meaning to diabetes management applied to other areas as well, such as when the young adults reflected on the life opportunities that arose from diabetes. It seemed that this attribution of meaning provided the emerging adults with a purpose that transcended their day‐to‐day struggling with blood glucoses and made it possible to keep going despite the Sisyphusian aspect of their diabetes management. The benefits from attributing meaning to diabetes management are also suggested in previous research, pointing to meaning in life as an important factor for psychological well‐being in people with a chronic illness.^32^

Making space for emotions was yet another key strategy to facilitate diabetes management. Throughout the blogs, diabetes management not only implied practical acts but also required that the emerging adults navigated emotional reactions on many levels, ranging from everyday frustrations about blood glucoses to serious anxiety and eating disorders. Concretely, the emerging adults benefitted from taking pauses, talking about emotional problems, taking a sick day, postponing an examination and seeking professional help. Accordingly, emerging adults wished support for emotional problems from peers and family, and especially from healthcare providers. This is crucial, considering that mental health problems double the odds of having poor glycaemic control.^33^

This study has several strengths. Blogs by emerging adults gave unique access to a diabetes population, not necessarily easy to recruit and potentially less open in a research interview or in a survey than on personal blogs. Likewise, the blogs proved a textually rich source of information and gave nuanced insight into everyday details that are easy to miss. Some of the findings resonated with previous findings, while some of the findings were specific to this study, and thus substantiate and expand the evidence base. Also, the study showed that blogs provided ample potential for future research in emerging adults.

Still, there were also potential limitations to consider. The blogs probably did not provide an unbiased route to young people's innermost perspectives. It is likely that peer readers (‘DOC’, the diabetes online community)—or family readers played a role for what was revealed online. Likewise, general norms for ‘online identity’ may have been at stake, such as norms for how to present one‐self online. For instance, it has been suggested that people tend to ‘overshare’ at the internet.^34^ Another potential limitation was that the bloggers were primarily female. This limitation is well‐known within qualitative health research, as males are more difficult to recruit than women.^35^ Methodologically, it is possible that other researchers would have angled themes differently, still thematic analysis is a well‐tested procedure and the themes identified in this study were compatible with broader theories on emerging adulthood and with themes identified in other qualitative studies of diabetes blogs.^16^, ^17^ Also, our pragmatic expectation was that saturation would be reached by including at least 15 blogs. However, the total number of identified blogs was 16, and we decided to include all. We considered that we had reached saturation regarding major themes, though clearly, we would have liked to sample more blogs written by male authors, but none were identified. Finally, we did not analyse in depth the full content of the blogs but focused on approximately one year. We reduced the risk of missed nuances by considering saturation, and for a minority of blogs to expand the timeframe.

This study implies that it seems worthwhile to further explore how psychological facilitators can be translated into practical clinical tools. For instance, it could be explored how belonging to a peer group—or other kinds of belonging—can be consistently supported, also for those who are not attracted to online fora. It could also be explored how to facilitate an individualized approach to manage and cope with fluctuating blood glucose and how to consistently address emotional problems. Likewise, it seems highly promising to find methods for involving family in diabetes care.

In conclusion, blog analysis is a novel approach to comprehend emerging adults with type 1 diabetes, and the blogs authored by emerging adults provided insight into psychological key facilitators for diabetes management in the challenging phase of emerging adulthood. On an overall note, the young people consistently strove to integrate diabetes in a sustainable way in their everyday lives. Significant strategies were to develop an individualized approach to manage and cope with fluctuating blood glucoses, to establish belonging to a group of diabetes peers to, to attribute a meaning to diabetes management that transcended day to day management and to making space for emotional reactions. Also, diabetes management was facilitated by emotional and caring backup from family and by support from health care providers who were perceived as nonjudgmental. Overall, this study underscores the need for understanding diabetes management in emerging adulthood as intertwined with the developmental features of the age.

## CONFLICT OF INTEREST

No conflicts of interest.

## AUTHORS' CONTRIBUTIONS

CBJ conceived the idea for the study, designed, and organized it, extracted and analysed data, and wrote the first draft of the paper. MJR, FP and HBN contributed to design, and analysis, and commented on drafts of the manuscript.

## ETHICAL APPROVAL

The blog data were publicly accessible and required no approval from ethics committee.

## Data Availability

The blog data were publicly accessible.
